# Synthesis and evaluation of nanosized aluminum MOF encapsulating Umbelliferon: assessing antioxidant, anti-inflammatory, and wound healing potential in an earthworm model

**DOI:** 10.1186/s12896-024-00889-8

**Published:** 2024-09-15

**Authors:** Rabab M. Thabit, Fatma El-Zahraa A. Abd El-Aziz, A. Abu El-Fadl, A. A. Abu-Sehly, Ahmed M. Sayed

**Affiliations:** 1 Physics Department, Faculty of Science, Assiut University, Assiut, 71516 Egypt; 2 Department of Zoology, Faculty of Science, Assiut University, Assiut, 71516 Egypt; 3 Chemistry Department, Faculty of Science, Assiut University, Assiut, 71516 Egypt

**Keywords:** Al-MOF, Umbelliferon, Drug delivery, Biological activities, Wound healing, Earthworms

## Abstract

Nanoporous aluminum metal–organic framework (Al-MOF) was synthesized via solvothermal methods and employed as a carrier matrix for in vitro drug delivery of Umbelliferon (Um). The encapsulated Um was gradually released over seven days at 37 °C, using simulated body fluid phosphate-buffered saline (PBS) at pH 7.4 as the release medium. The drug release profile suggests the potential of Al-MOF nanoparticles as effective drug delivery carriers. Structural and chemical analyses of Um-loaded Al-MOF nanoparticles (Um-Al MOF) were conducted using Fourier-transform infrared (FTIR) spectroscopy, X-ray diffractometry (XRD), and ultraviolet–visible (UV–Vis) spectroscopy. Thermal gravimetric analysis (TGA) was employed to investigate the thermal stability of the Al-MOF nanoparticles, while Transmission Electron Microscopy (TEM) was utilized to assess their morphological features. Um-Al MOF nanoparticles demonstrated notable antioxidant and anti-inflammatory properties compared to Um and Al-MOF nanoparticles individually. Moreover, they exhibited significant enhancement in wound healing in an earthworm model. These findings underscore the potential of Al-MOF nanoparticles as a promising drug delivery system, necessitating further investigations to explore their clinical applicability.

## Introduction

Porous nanoscale metal–organic frameworks (nanoMOFs) have recently gained attention in the biomedical field due to their amphiphilic interior milieu, ideal for the adsorption of diverse guests such as drugs, cosmetics, and biological gases [1–6]. MOFs are prepared through the self-assembly of a metal and an organic linker [7]. The porous hybrid organic–inorganic materials have various applications due to their unique properties [1]. The physicochemical features of nanoMOFs, including composition, molecular weight, size, and surface chemistry, significantly influence their performance in terms of biocompatibility, biodegradability, biodistribution, and efficacy [8–13]. Multifunctional TiO_2_/C nanosheets derived from 3D MOFs were developed for use in advanced cancer therapy. These nanosheets combine several therapeutic modalities, including sonodynamic therapy (SDT), photothermal therapy (PTT), and chemodynamic therapy (CDT), which are effective at mild temperatures. The TiO_2_/C nanosheets are designed to enhance treatment efficiency while minimizing damage to surrounding healthy tissues [14]. Also, a core–shell CuO/C material derived from MOFs was developed for the real-time analysis of glucose [15]. Interestingly, a novel wound dressing was synthesized by covalently grafting a polycation onto bacterial cellulose to create a material with enhanced antibacterial and anti-cell adhesive properties [16].

Aluminum metal–organic framework (Al-MOF) has demonstrated significant promise as heterogeneous catalysts in various chemical reactions [17]. It has also been studied for its potential in energy storage applications, specifically in supercapacitors and batteries [18]. Additionally, Al-MOFs have been explored for their ability to purify and remove contaminants, due to their adsorption properties [19]. Al-MOF nanoparticle is one of the most promising nanocarriers due to its remarkable ability to load various therapeutic compounds and regulated release profiles that enhance the bioavailability of the drug [20–23]. Al-MOF created effectively multifunctional materials that combine drug delivery and imaging, offering significant potential in biomedical applications. The aluminum ions help form a stable framework with the necessary hierarchical pore structure, which is essential for both efficient drug loading and controlled release. Additionally, the Al-MOF's properties contribute to the material’s fluorescent capabilities, making it useful for imaging applications [24]. Al-MOF nanoparticles can form one- or two-dimensional inorganic sub-networks through the connecting of aluminium-centered octahedra [25–27]. The Al-MOF nanoparticles can reversibly absorb and release water, and they typically show exceptional thermal stability up to 450 °C. These characteristics make Al-MOFs more stable than other MOFs that are extremely sensitive to moisture. The strong Al–O bonds and aluminum's octahedral coordination give this exceptional hydrothermal stability [26, 27]. Therefore, many studies utilized these unique properties of Al-MOF nanoparticles in various applications.

Umbelliferon (Um) is known as 7-hydroxycoumarin widely distributed in plants and classified as a benzopyron in nature [28–37]. Um showed various activities including anti-diabetes, anti-cancer, anti-infection, anti-rheumatoid arthritis, neuroprotection, and reduction of tissue damage in the liver, kidneys, and heart [28, 29, 31]. Um regulates blood glucose, lipid metabolism, improving insulin resistance, cardiac hypertrophy, tissue fibrosis, control of oxidative stress, inflammation, and apoptosis [38–42]. The main regulatory action of Um is to mitigate inflammation and oxidative stress [31].

In this study, Um was loaded successfully into Al-MOF nanoparticles to evaluate its antioxidant, anti-inflammatory, and wound-healing properties. Also, the Um release profile was analyzed over seven days in a mimicked body fluid solution of phosphate-buffered saline (PBS) at pH 7.4. The synthesized Al-MOF nanoparticles were characterized by thermal gravimetric analysis (TGA), X-ray diffractometry (X-ray), ultraviolet–visible spectrum (UV–Vis), and Fourier-transformed infrared spectroscopy (FTIR). Morphological features were investigated by Transmission Electron Microscopy (TEM). The antioxidant activity of Um-Al MOF nanoparticles was estimated in vitro through a free radical scavenging activity test (Scavenging of DPPH radical), and the anti-inflammatory activity was determined by measuring the protein expression levels of NF-kB, IL6, and TNFα using ELISA. Moreover, we assessed the wound-healing properties of Um-Al MOF nanoparticles in earthworms as a feasible alternative model for human skin because they both possess similar triterpene and triterene sterols [43–46]. Also, there are similarities in many biomolecules, such as 7-dihydroxycholesterol and cholestatrin-3b-ol (9-DDHC) between earthworms and mammalian skins [44]. Earthworms have long been known to be a major component of the soil environment [47]. It constitutes about 80% of the total soil biomass Invertebrates in an ecosystem [48]. These results suggest that incorporating Um into Al-MOF nanoparticles effectively enhances its bioavailability and bioactivity.

## Materials and methods

### Drugs and chemicals

Aluminium Nitrate Nonahydrate (Al(No_3_)_3_⋅9H_2_O, 98%) (375.13 g/mol), Organic linker 1,4-benzene dicarboxylate (BDC) (166.13 g/mol), Dimethylformamide (DMF, 98%), Um (162.14 g/mol) and Ethanol (C_2_H_6_O, 0.95%) were purchased from (Sigma).

### Preparation of Al-MOF nanoparticles and Um-Al MOF nanoparticles

Preparation of Al-bdc metal–organic framework by hydro/solvothermal synthesis in a steel pressure vessel by direct combining of reactants (metal salt and organic linkers) into Dimethylformamide (DMF) as a solvent and under solvothermal conditions for 12 h at 120 $^\circ C$ and then vessel cooled for another 12 h at room temperature [49]. One ml of Al-MOF ethanol–water solution was added to one ml Um ethanol–water in 5:2 molar ratio to form Um-Al MOF nanoparticles. Then followed by centrifuging of Um-Al MOF nanoparticles for 30 min at 8000 rpm.

### Characterization of Um-Al MOF nanoparticles

#### Measurement of drug entrapment efficiency (EE) % and drug loading (LC) capacity

The EE and LC of Al-MOF for Um were examined by separating Um-Al MOF nanoparticles from an aqueous medium containing free drug after 30 min of centrifugation at room temperature with an 8,000 rpm. A UV spectrophotometer set to 304 nm was used to measure the amount of free Um against the standard curve of Um. The EE and LC were calculated according to the following Eqs. (1) and (2)

1 $$EE\left(\%\right)=\frac{\text{total amount of umbelliferrone}-free\;\text{umbelliferrone}}{\text{total amount of umbelliferrone}}x100\%$$

2 $$LC\left(\%\right)=\frac{\text{total amount of umbelliferrone}-free\;\text{umbelliferrone}}{\text{nanoparticles weight}}x100\%$$

#### Fourier transform infrared spectroscopy (FT-IR)

Um, Al-MOF and Um-Al MOF FT-IR spectra were obtained using FT-IR Spectrophotometer (Thermo Scientific, USA) using smart OMNI-sampler Accessory. Separately, 400–4000 cm^−1^-sized pelletized discs comprising 2 mg of each specimen and 10 mg of potassium bromide were examined.

### X-ray diffractometry (XRD)

On the PW 1710 control unit Philips XRD (Holland), the X-ray diffraction patterns of Um, Al-MOF, and Um-Al MOF were recorded. This was done while operating at 40 kV voltage and 30 mA current with a wavelength of (λ = 1.5405 Å) and a scanning variation of 4° to 80° at 2θ angle with a step size of 0.06°, the samples were scanned using graphite monochromatized high-intensity Cu Kα X-radiation.

#### Transmission Electron microscopy (TEM)

The specimens' morphological features were directly obtained by using TEM (Jeol 2100). Samples were prepared by dissolving them in ethanol, then sonicating them for ten minutes, applying them on a cupper grid, and letting them air dried at room temperature. Once the sample had completely dried at room temperature, the images were taken and analyzed using Soft Imaging Viewer and Digital Micrograph software.

#### Thermal gravimetric analysis (TGA)

Thermogravimetric analysis was utilized to describe the specimens. The study was performed using a Shimadzu DTG-60H simultaneous DTA-TG apparatus in the 50–600 °C temperature range, with a heating rate of 10 °C min^–1^ and a flow rate of 40 ml min^–1^ in an air-filled atmosphere.

#### In vitro drug release study

By shaking the samples in an incubator, they were suspended in PBS (pH 7.4). (37 °C,150 rpm). Supernatants were collected (3000 rpm, 15 min) at different time points (1, 2, 4, 6, 24, 48, 72, 96, 120, 144, 168 h) to calculate the amount of released Um. PBS was used as the solvent to obtain Um's standard curve, and a UV spectrophotometer was used to measure the absorbances at 304 nm.

### Measurement of antioxidant activity

The ability of free Um and Um-Al MOF nanoparticles to scavenge the stable nitrogen-centered free radical DPPH (2,2-diphenyl-1-picrylhydrazyl) was used to measure their antioxidant activity. Each sample (25 μl) was mixed with 2 ml of DPPH solution dissolved in methanol at a concentration of 25 μM and incubated at room temperature for 30 min in the dark. The absorbance was then measured at 517 nm against a methanol control. Factors such as the inherent antioxidant activity/radical scavenging activity, sample concentration, and degree of decolorization of the violet color of the DPPH methanolic solution were taken into account. Antioxidant substances can either directly reduce the DPPH radical by donating electrons or quench it by providing hydrogen atoms. The scavenging ability of each sample was expressed as a percentage of antioxidant activity (AA), calculated using the formula below, where A represents absorbance:

$$AA=(\frac{\text{A DPPH },517\text{nm }- A samples ,517nm}{\text{A DPPH },517\text{nm}}) x100\%$$

Trolox served as the reference standard for determining the Trolox Equivalent Antioxidant Capacity (TEAC) values, and a calibration curve was generated across a range of concentrations (10–250 μg/ml). The results were reported in Trolox equivalents per milliliter of solution. TEAC was employed to quantify the antioxidant specimens' efficacy in scavenging DPPH radicals relative to Trolox; higher TEAC values indicate the superior scavenging capacity of the specimens [50].

### Measurement of anti-inflammatory activity

#### Determination of the protein expression levels of COX2, IL6, NF-kB p65 and TNFα using enzyme-linked immunosorbent assay (ELISA)

Protein expression levels of COX2, IL6, NF-kB p65, and TNFα were determined using enzyme-linked immunosorbent assay (ELISA) kits, with each assay performed in triplicate. Briefly, BV2 microglial cells (1 × 10^5) were seeded into 96-well plates and incubated overnight. The following day, cells were pretreated with or without specified concentrations of free Um, Al-MOF nanoparticles, and Um-Al MOF nanoparticles for 1 h before exposure to 0.1 μg/mL LPS for an additional 24 h. Post-incubation, 100 μL of culture medium supernatant was collected from each well, and the concentrations of COX2, IL6, NF-kB p65, and TNFα were quantified using ELISA kits.

### Earthworms experiments

#### In vivo evaluation of wound healing properties of Um-Al MOF nanoparticles in earthworm model

Earthworms were collected from the Assiut University farm and then transported to the Zoology Department laboratory for identification and separation. These worms underwent a two-week adjustment period to acclimate to controlled laboratory conditions, the temperature between 25–28°C during a 12-h day-night cycle. The worms were placed in plastic containers filled with moist soil, supplemented with dried livestock manure to serve as their food. To begin the experiment, sterile medical scalpels were used to make tiny cuts in the earthworms' skin.

#### Treatment of earthworms

Earthworms were divided into five groups; each group contained (15 worms of the same weight and age) as the flowing.

- Group I: (control) remained normal without cut.
- Group II or untreated: received Vaseline only.
- Group III: received a mix of Vaseline and Um-Al MOF,
- Group IV: received a mix of Vaseline and Um.
- Group V: received a mix of Vaseline and Al-MOF.

All the treated worms were anesthetized in 5% ethanol and subjected to a cut in the skin (the three layers; epidermis, circular muscle, and longitudinal muscle of the anterior part (first segments) with a sterile scalpel, making a wound. 1 mg of Um and its corresponding concentration of Um-Al MOF and Al-MOF were suspended in 100mg Vaseline (1%) to be applied topically to the wounds. Treatment was performed three times daily regularly for 17 days and the wound area was uncovered according to [43]. The worms were preserved in A petri dish covered with filter paper is moistened to achieve the desired humidity level. The wound area was estimated daily and measured millimeters by macroscopic observation. The protocol/experiments have been approved by the Ethics Committee for Animal Experimentation, Faculty of Science, Assiut University, Egypt (no.01/2024/0008).

#### Histological investigations

Tissue Sects. (5 μm) from different groups of earthworms were mounted on slides and dried overnight at 37 °C, de-waxed in xylene and hydrated in a graded series of alcohols and stained by Mason's trichrome [51].

#### Ultrastructural study

##### **Preparation for semithin sections**

Worms from different groups were washed in phosphate buffer (pH 7.2), 3–4 times for 20 min each. After fixation in 4% cold glutaraldehyde, the samples were examined. They were then fixed in 1% osmium tetroxide (OsO4) for 2 h, then washed 4 times. And dried in an ethyl alcohol series of increasing concentration. To remove alcohol residue, worm samples are embedded for 30 min in propylene oxide, then embedded it in propylene oxide plus Epon 812 (1:1, v/v) for 30 min. Finally, they were embedded in Epon 812 for 4 h. Place in the oven for two days at 60°C. Then prepare semithin sections with a thickness of 0.5 μm using LKB ultra-microtome and then stain with toluidine blue [52].

##### **Preparation for transmission electron microscopy (TEM)**

Semithin sections of worms were evaluated for localization of the worms' tissues and accordingly, ultrathin sections were prepared. Ultrathin Sects. (50–80 nm) were cut using Leica AG ultramicrotome, and stained with uranyl acetate and lead citrate [53]. Sections were examined under TEM (Jeol, 100 CXII) operated at 80 kV. Electron micrographs were taken for selected semithin regions, reconstructed, and processed by a photoshop computer program to study the selected samples. The results were obtainable as micrographs.

### Statistical analysis

The data were presented as mean ± SE. Statistical analyses were conducted using column statistics and one-way analysis of variance, followed by the Newman–Keuls multiple comparison test as a posttest. The computations were performed using the Prism program for Windows, version 5.0, developed by GraphPad Software Inc., San Diego, California, USA. Significance levels were set at *P* < 0.05, 0.01, or 0.001, considering differences both within and among the groups.

## Results

### EE and LC %

Using a UV spectrophotometer (PE, USA) adjusted at 324 nm, the EE and LC of Um-Al MOF nanoparticles were found to be 90% and 18%, respectively. These results show the potential of Al-MOF nanoparticles as an efficient drug carrier.

### Fourier Transform Infrared Spectroscopy (FT-IR)

**Fig. 1:**
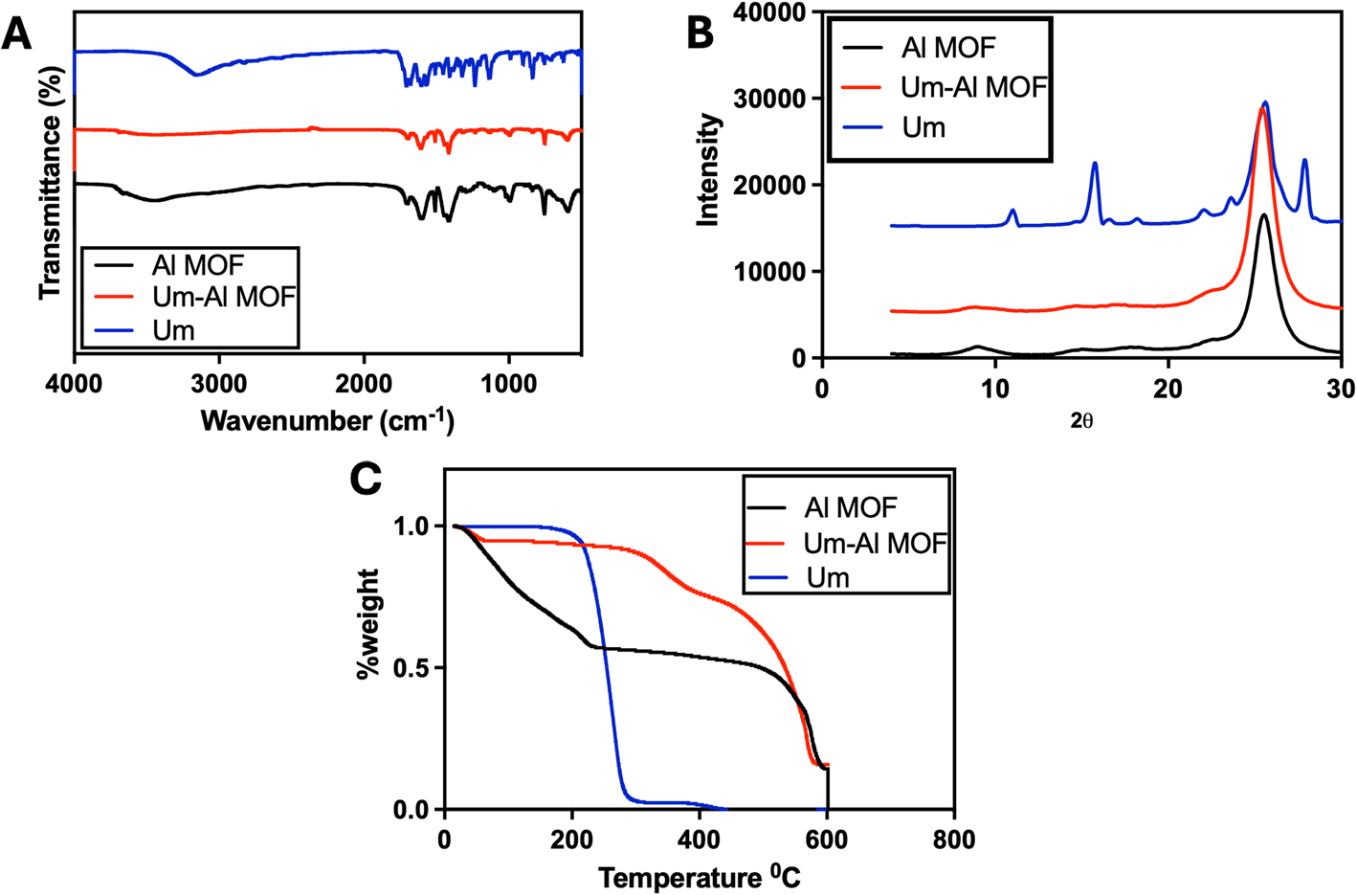
FTIR, XRD, and TGA analysis of the prepared nanoparticles. **A** FTIR spectra. **B** XRD analysis. **C** TGA analysis of Al-MOF nanoparticles, Um and Um-Al MOF nanoparticles composites prepared

The FT-IR spectra of Al-MOF nanoparticles and their components are presented in (Fig. 1A). Um's FT-IR spectrum displayed a sharp peak of the OH group at 3,153 cm^−1^. C-H stretching vibrations are identified at 3,122 and 2,987 cm^−1^, single bonded carbonyl group vibrations at 1388, 1,345, and 1,233 cm^−1^, and C–C stretching vibrations at 1,578, 1,454, 1,446, 1,409, and 1,386 cm^−1^, similar observation has been recorded previously [54]. The Fourier transform infrared spectra of the Al-MOF nanoparticles (Fig. 1A) show vibration bands in the range of 1,400–1,700 cm^−1^ for the carboxylic functional group. Additionally, bands at 1,608 and 1,512 cm^−1^ indicate asymmetric stretching (-COO-), whereas bands at 1,435 and 1,417 cm^−1^ are indicative of symmetric stretching (-COO-). Also, The absorption band at 1,669 cm^−1^ was caused by unreacted BDC molecules' –COOH group being trapped in Al-MOF nanoparticles’s cavities that agree with previously reported data [55]. The spectra of the Um-Al MOF nanoparticles showed changes at different peaks, with the characteristic peaks of Um disappearing in the Al-MOF nanoparticles spectrum, indicating that Um was impeded in the nanoparticles matrix rather than on the surface, suggesting a possible interaction between Um and Al-MOF nanoparticles.

### X-ray diffractometry (XRD)

The XRD patterns of Um, Al-MOF nanoparticles, and Um-Al MOF nanoparticles demonstrated the high crystallinity of the prepared Al-MOF nanoparticles (Fig. 1B). For the XRD pattern of Um, two strong peaks were found with diffraction peaks at 2θ of 11.2°, 16.0°, revealing the drug's crystalline nature (crystallinity percent = 92.2%) where the Joint Committee on Powder Diffraction Standards (JCPDS) card number for Um is 01–084-4965 [54]. Prominent diffraction peaks at 2θ of 8.5 and 16° indicated the crystallinity of Al-MOF nanoparticles (crystallinity percent = 51.6%) and JCPDS card number is 00–021-1919, 00–002-0173 [56]. The XRD pattern of Um-Al MOF nanoparticles showed some changes, where a decrement in the intensity of the diffraction peak at 8.66 and a shift in the diffraction peak at 12.4 were observed, suggesting the conjugation of Um to Al-MOF nanoparticles (crystallinity percent = 55.5%).

### Thermal gravimetric analysis (TGA)

The reduction of a defined mass of Al-MOF nanoparticles loaded with Um was studied using TGA by heating under air atmosphere flow from 50 to 600 °C at a constant rate of 10 °C/min and a flow rate of 40 ml/min. As shown in (Fig. 1C), TGA profiles of Al-MOF nanoparticles exhibited three weight losses within the range of 14.89–218.81 °C, 226.86–456.1 °C, and 464.24–585.93 °C, representing the thermal breakdown of Al-MOF nanoparticles and the evaporation of physically adsorbed water. TGA of Um showed three stages of weight loss, corresponding to the degradation temperature in the range of 14.89–173.38 °C (physically adsorbed water and volatile solvents loss), 184.06–279.35 °C, and 282.47–400.17 °C (thermal decomposition of Um). Um-Al MOF nanoparticles conjugate displayed three thermal breakdowns in the range of 14.89–301.34 °C, 309.4–418.82 °C, and 432.15–574.18 °C, respectively, due to the degradation of Um-Al MOF nanoparticles conjugate and water content. Um was successfully encapsulated into Al-MOF nanoparticles, as demonstrated by the thermogravimetric analysis (TGA) of the Um-Al-MOF conjugate, which exhibited the thermal degradation profiles characteristic of both Um and the Al-MOF nanoparticles.

### Transmission electron microscopy (TEM)

**Fig. 2:**
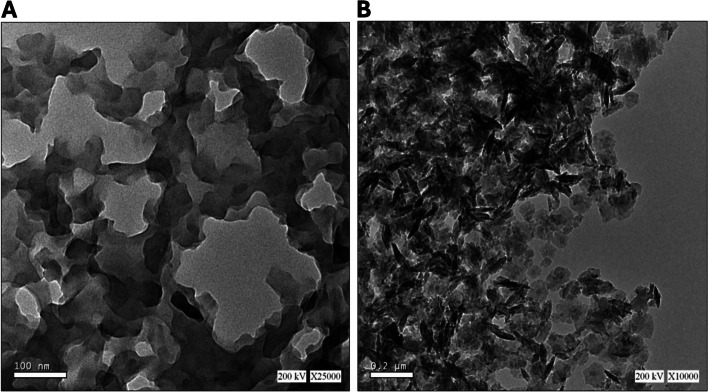
Transmission electron microscopy (TEM) images OF (**a**) Al-MOF nanoparticles, (**b**) morphology and dispersity of Um-Al MOF nanoparticles

Figure 2 presents transmission electron micrographs of Um-Al MOF and Al-MOF nanoparticles. In Fig. 2A, Al-MOF nanoparticles were morphologically analyzed and found to be grey, light spherical, polydisperse, smooth-surfaced, and around 100 nm. In Fig. 2B. Um-Al MOF nanoparticles, with sizes around 200 nm, seemed almost more aggregated. Um can engage in various interactions with Al-MOF, including hydrophobic interactions, π-π stacking between Um's aromatic ring and the conjugated systems within Al-MOF, as well as electrostatic interactions involving Um's hydroxyl (-OH) and carbonyl (C = O) functional groups with the charged functional groups on the surface of Al-MOF nanoparticles. These interactions contribute to an increased propensity for the particles to aggregate or form clusters, rather than staying evenly dispersed. The grey Al-MOF changed to a dark color as a result of saturation with the loaded Um.

### In-vitro drug release study

**Fig. 3:**
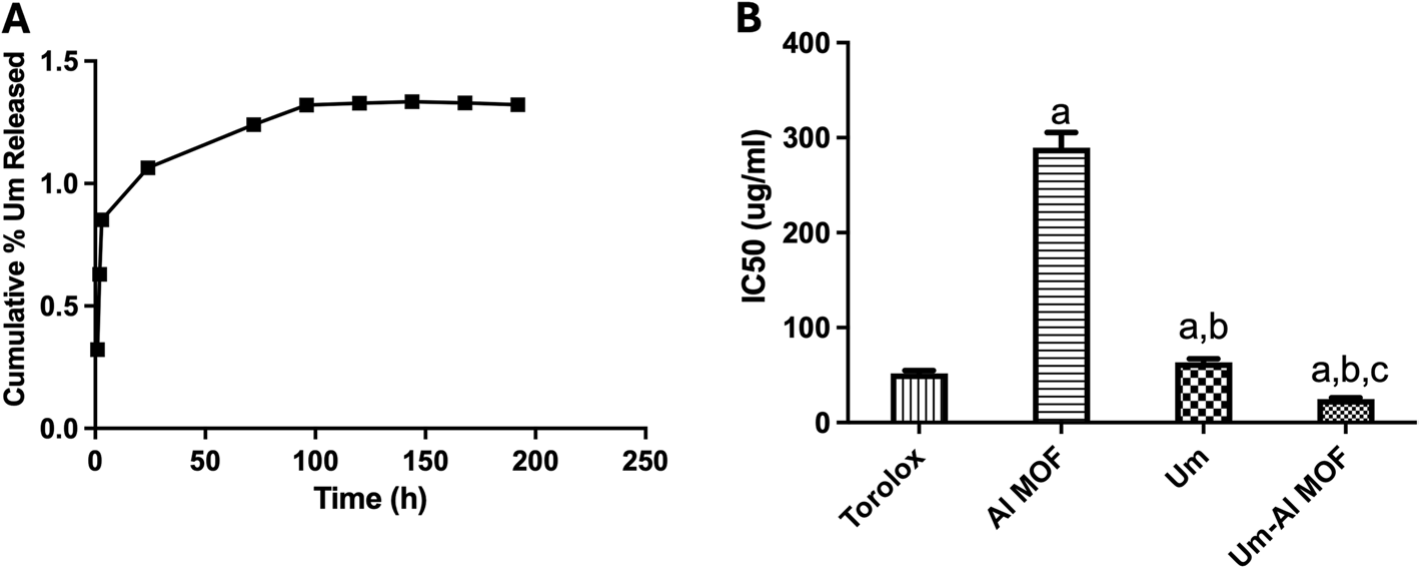
**A** The in vitro release profile of Um from Al-MOF nanoparticles. **B** DPPH% scavenging activity of Al-MOF nanoparticles, Um, and conjugated Um-Al MOF nanoparticles represented by IC50 values. The results were presented as a mean and SEM. **a** significant versus “control”. **b** significant versus “Al-MOF”, **c** significant versus “Um” at *p* < 0.05

Um in-vitro release profile via Al-MOF nanoparticles in PBS at physiological pH 7.4 at 37 °C is displayed in (Fig. 3A). The release was initially fast and continued for more than 7 days, indicating that Al-MOF nanoparticles achieved a sustained drug release pattern. The biphasic drug release profile, consisting of an initial burst release followed by a slower, sustained release phase, is influenced by the distinct interaction of Um molecules with the Al-MOF structure. During the initial burst release phase, Um molecules loosely associated with the surface of the Al-MOF rapidly dissolve into the surrounding medium. These surface-bound molecules are not fully encapsulated within the Al-MOF, resulting in their quicker release. In contrast, the slower sustained release phase occurs as Um molecules that are more deeply integrated within the pores of the Al-MOF gradually diffuse out, taking more time to be released compared to those on the surface. The slow and small release confirmed the perfect complexation of Um and Al-MOF nanoparticles.

### Antioxidant activity of the formulation

Measurement of Antioxidant Activity Exogenous factors and agents, as well as normal metabolic processes, generate free radicals, which can quickly initiate the peroxidation of membrane lipids, resulting in the accumulation of lipid peroxides. A hydrogen free radical is present in DPPH, which exhibits a distinctive absorption at 517 nm. The DPPH test, measuring the decrease of the DPPH radical in the presence of an antioxidant molecule, was used to assess the Um formulations' in-vitro antioxidant activity. The proton scavenging activity of Al-MOF nanoparticles, both free and loaded with Um, was determined using DPPH in this study. The total DPPH scavenging potential of Al-MOF nanoparticles and Um-Al MOF nanoparticles at various concentrations were determined, and the results are shown in (Fig. 3B). Um exhibited its well-known antioxidant power, scavenging the DPPH radical. Although Al-MOF nanoparticles showed weak antioxidant activity, the conjugation of Um into Al-MOF nanoparticles showed almost complete scavenging of the DPPH radical, indicating enhanced antioxidant properties of Um upon conjugation into Al-MOF nanoparticles.

### Anti-inflammatory activity of the formulations

**Fig. 4:**
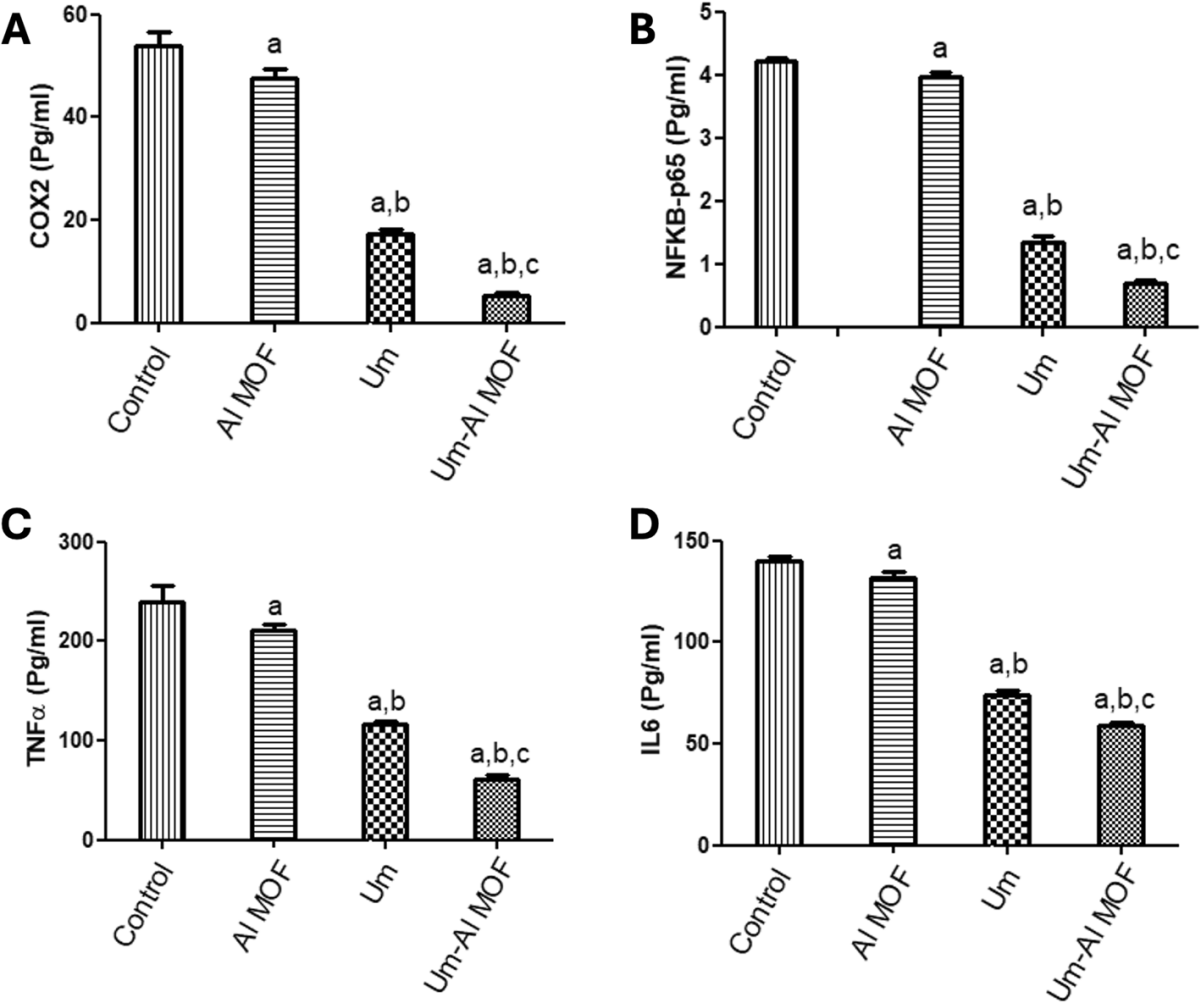
Anti-inflammatory biomarkers expression levels in LPS-stimulated microglial BV2 cells using ELISA. **A** COX2. **B** NFkB. **C** TNFα. **D** (IL6). The results were presented as a mean and SEM. **a** significant versus “control”. **b** significant versus “Al-MOF”, **c** significant versus “Um” at *p* < 0.05

Inhibition of LPS-induced Expression of COX2, IL6, NF-kB p65, and TNFα According to studies, LPS increases the generation of ROS in BV2 cells, which may cause harm to many cellular structures and functional molecules. Additionally, ROS functions as a messenger molecule in cells, triggering signaling pathways and transcription factors like COX2, IL6, NF-kB p65, and TNFα. To elucidate the anti-inflammatory action of Al-MOF nanoparticles, free and Um-Al MOF nanoparticles, the effect on the production of ROS was first evaluated. ELISA was used to validate the protein expression of COX2, IL6, NF-kB p65, and TNFα in LPS-stimulated microglial BV2 cells. ELISA analysis revealed that LPS significantly elevated COX2 expression levels (54.02 ± 2.47), IL6 (140 ± 2.14), NF-κB (4.21 ± 0.06), and TNFα (239.4 ± 16.1) compared to the control group. However, pretreatment with free Um significantly inhibited the increased expression of COX2 (17.23 ± 0.92 vs 54.02 ± 2.47). Although pretreatment with Al-MOF nanoparticles did not affect the increased expression of COX2 (47.59 ± 1.84 vs 54.02 ± 2.47), the conjugated Um exhibited the highest potential inhibitory activity for the increased expression of COX2 (5.45 ± 0.34 vs 54.02 ± 2.47) (Fig. 4A). Similarly, pretreatment with Al-MOF nanoparticles significantly inhibited the increased expression of NF- κB p65 (3.97 ± 0.07 vs 4.21 ± 0.06), free Um (1.34 ± 0.09 vs 4.21 ± 0.06), and conjugate (0.69 ± 0.04 vs 4.21 ± 0.06) (Fig. 4B). Additionally, pretreatment with Al-MOF nanoparticles significantly inhibited the increased expression of TNFα (210.4 ± 6.15 vs 239.4 ± 16.1), free Um (116 ± 3.62 vs 239.4 ± 16.1), and conjugate (61.38 ± 3.51 vs 239.4 ± 16.1) (Fig. 4C). Furthermore, pretreatment with Al-MOF nanoparticles inhibited the increased expression of IL6 (131.5 ± 3.1 vs 140 ± 2.14), free Um (73.96 ± 2.09 vs 140 ± 2.14), and conjugate (59.1 ± 1.72 vs 140 ± 2.14) (Fig. 4D). Notably, the conjugated Um (Um-Al MOF nanoparticles) exhibited the highest potential inhibitory activity for the increased expression of COX2, IL6, NF-kB p65, and TNFα signaling cascades. These results demonstrated that the inclusion of Um in Al-MOF nanoparticles may be useful in improving the bioavailability and bioactivity of Um as an anti-inflammatory agent by inhibiting the stimulation of COX2, IL6, NF-kB p65, and TNFα pathways.

### In vivowound healing effect of Um-Al MOF in earthworm's skin model

**Fig. 5:**
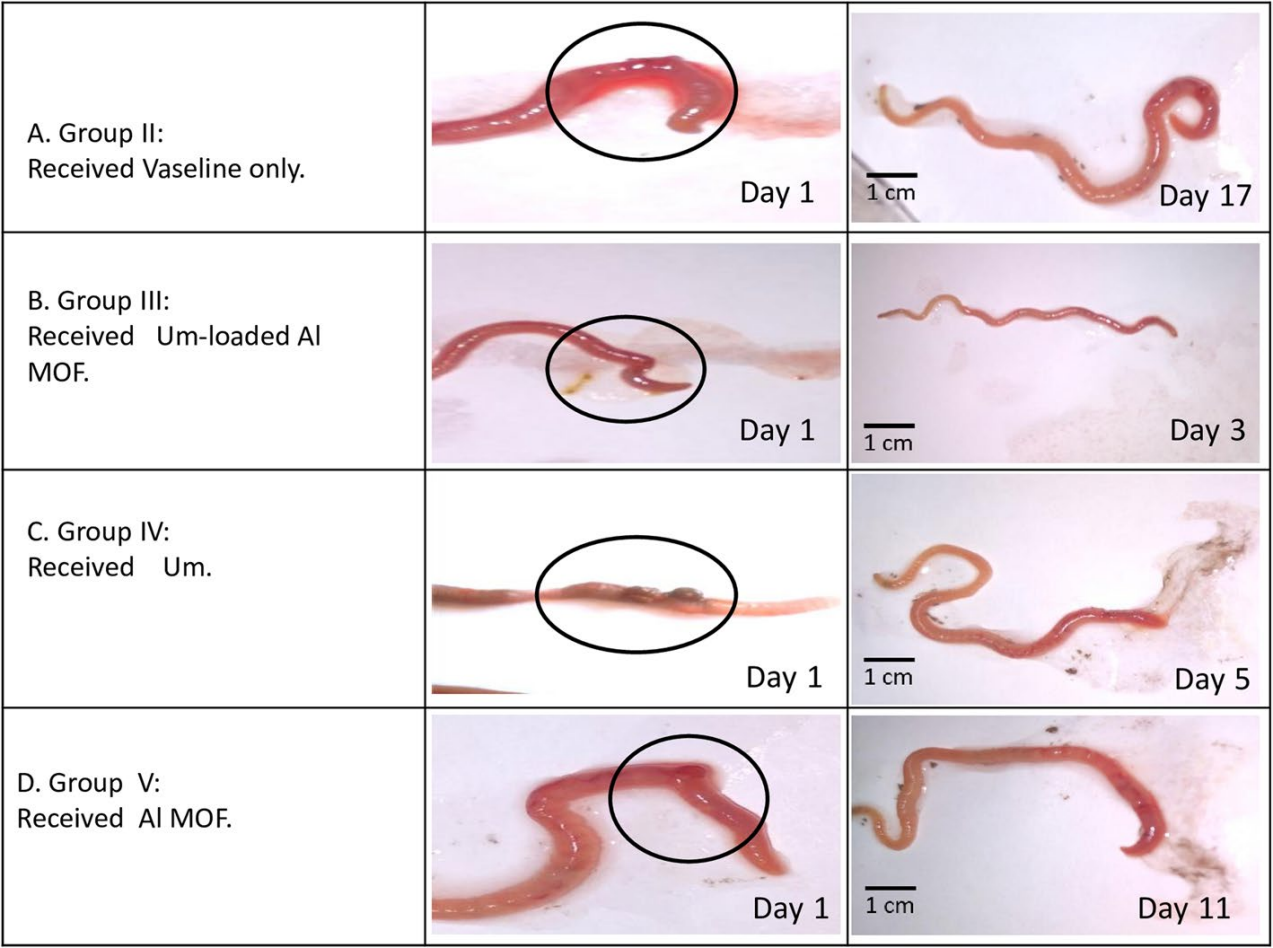
Macroscopic observation of the different groups of earthworms (*Allolobophora caliginosa*) after induction of surgical wounds and examination of wound healing; (**A**) worms were left untreated to the mercy of natural healing by Vaseline; (**B**) worms received Um-Al MOF; (**C**) worms received Um and (**D**) worms received Al-MOF, cut in the anterior part (circle))

**Fig. 6:**
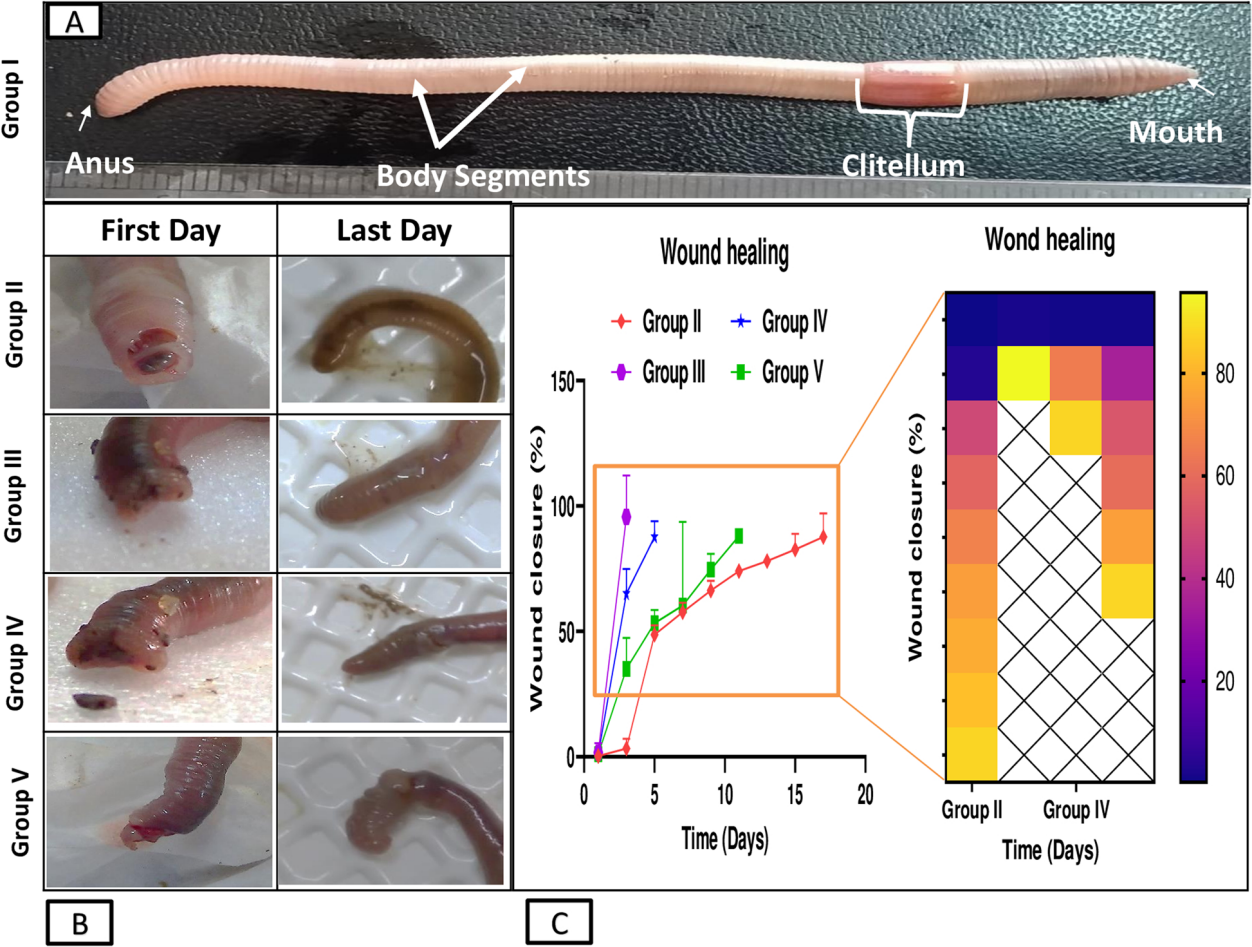
Macroscopic observation of the different groups of earthworms (*Allolobophora caliginosa*); (**A**) normal earthworm group I, (**B**) treated groups II, III, IV and V, (**C**) Differences among groups regarding wound closure at different experimental periods

This study demonstrates significant progress in the wound healing process using Um-Al MOF. Importantly, no worm fatalities were observed during the experiment. On the first day after injury, groups II, III, IV, and V exhibited hemorrhage, edema, redness, and exudation around the wound area. Following treatment, group III showed the fastest coagulation, with complete wound closure occurring within 3 days, compared to 17 days in the untreated group II, nearly 5 days in group IV treated with Um, and over 11 days in group V treated with Al-MOF (Fig. 5). Analysis of data from a representative experiment revealed that on the first day post-wounding, the wound diameters were similar across all groups. However, by day 3 post-wounding, worms treated with Um-Al MOF nanoparticles achieved 100% wound closure (Fig. 6).

#### Histological observation

**Fig. 7:**
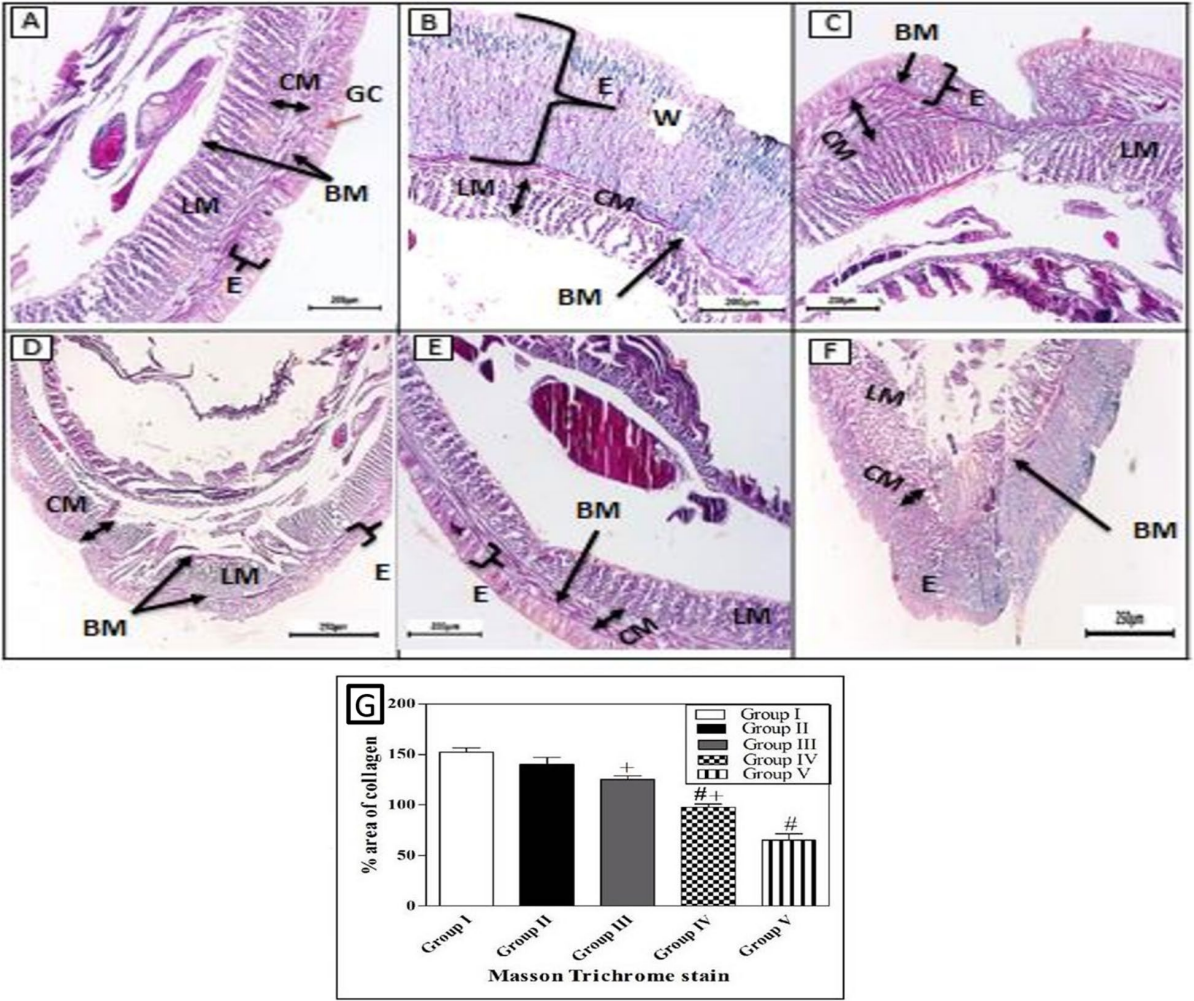
Photomicrographs of the cross section of the different groups of earthworms (*Allolobophora caliginosa*) after induction of surgical wounds and examination of wound healing; (**A**) Group I: normal earthworm; (**B**) worm on the first day of injury, (**C**) Group II; worms received Vaseline; (**D**) Group III; worms received Um-loaded Al MOF, (**E**) Group IV; worms received Um, (**F**) Group V worms received Al MOF, and (**G**) Differences among groups regarding % area of collagen deposition at different experimental periods. (Masson's trichrome stain) The collected data from 10 individual earthworm in each group for quantitative deposition of collagen. + *P* < 0.05, Control vs. Um-loaded Al MOF, # + *P* < 0.05 Control vs. Um, # *P* < 0.05, Control vs. Al MOF group. ((BM); basal membrane, (CM); circular muscle layer, (E); epidermis, (GC); gland cells, (LM); longitudinal muscle layer, (W);Wound)

When comparing the treated groups with the natural structure of the earthworm (*Allolobophora caliginosa*); (group I), it appears on the surface that they are composed of the epidermis, among these columnar cells, various gland cells, and beneath them are two muscular layers; the circular muscles, then the longitudinal muscles. It was observed that all groups that received an injury had all the symptoms of inflammation preserved on the first day of the induced injury. Including redness, bleeding, edema, and exudate around the wound area. The skin of earthworms, A. *caligino*sa on the third day after injury, Masson's trichrome staining showed in group III, transverse section passing through the body wall showed the normal structure of epidermal, circular, and longitudinal muscles. The transverse section of group II, V of *A. caliginosa* demonstrated vacuolization, and hypertrophy of epithelial cells, illustrating degeneration of circular with a large amount of inflammatory cell infiltration and longitudinal muscles in addition to vacuolization and hypertrophy of cells. The transverse section of group IV of *A. caliginosa* inflammatory cells was almost few, the body wall showed the structure of epidermal, circular, and longitudinal muscles. However, there are gaps and disintegration in the installation (Fig. 7). However, from the 3rd to the 17th day post-treatment, a noticeable reduction in collagen deposition was observed in the groups treated with Um and Al MOF, compared to the group treated with Um-Al MOF. The treatment with Um-Al MOF significantly enhanced collagen deposition in the earthworms. Collagen deposition was quantified using ImageJ software, with data collected from three worms in each group. The treatment with Um-Al MOF resulted in collagen levels comparable to those in the control group (Fig. 7, G).

#### Electron microscopic observation

**Fig. 8:**
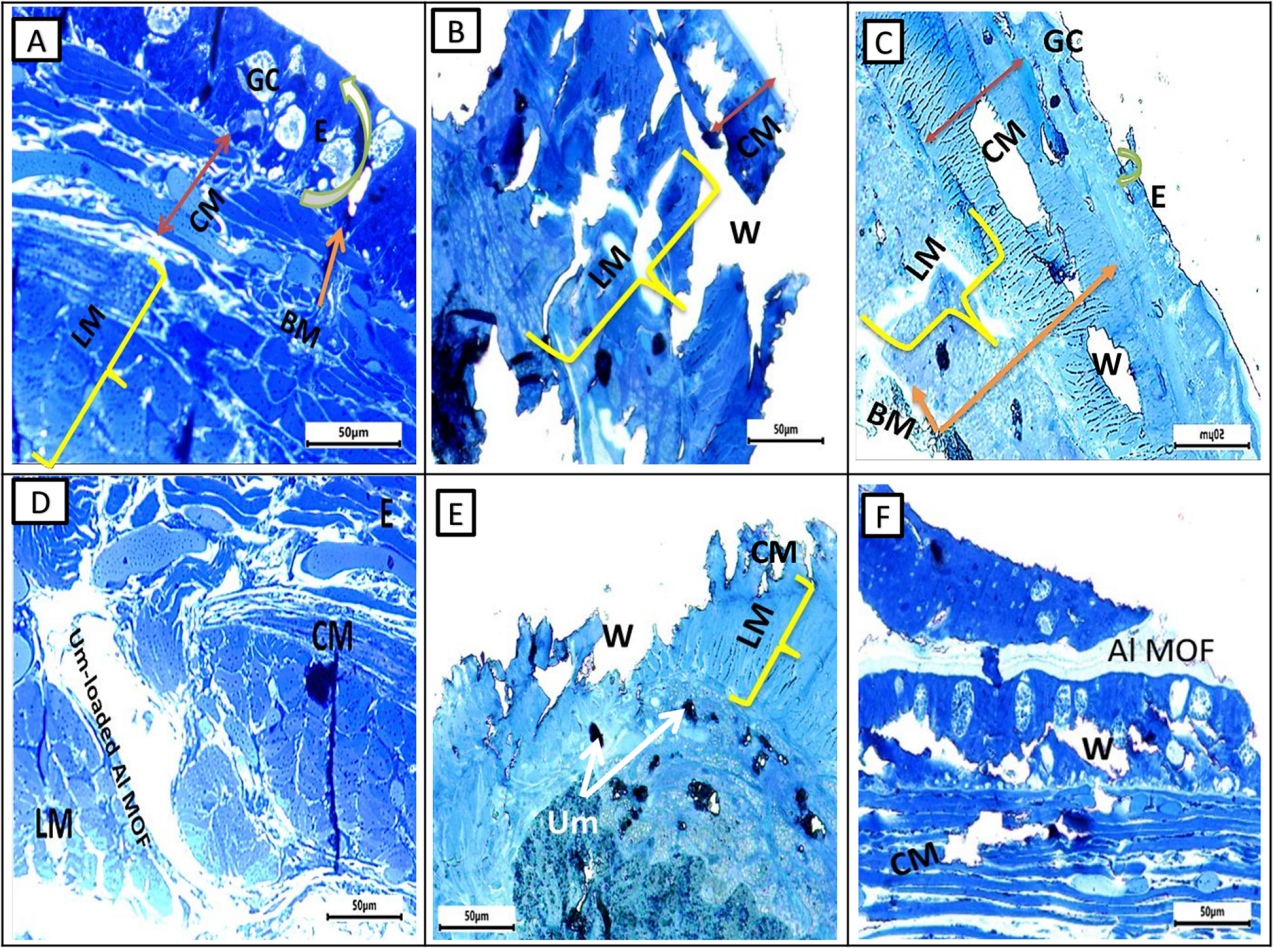
Photomicrographs of semithin sections of the different groups of earthworms (*Allolobophora caliginosa*) after induction of surgical wounds and examination of wound healing; (**A**) Group I: normal earthworm; (**B**) worm on the first day of injury, (**C**) Group II; worms received Vaseline; (**D**) Group III; worms received Um-Al MOF, (**E**) Group IV; worms received Um, and (**F**) Group V worms received Al-MOF. (Toluidine blue stain). ((BM); basal membrane, (CM); circular muscle layer, (E); epidermis, (GC); gland cells,, (LM); longitudinal muscle layer,(W);Wound)

Photomicrograph of semithin section of earthworms, *A. caliginosa* from all groups on the first day displayed a loss of structure, an inclination toward excessive glandular epithelium, and the destruction of the ectodermal layer and cuticular membrane. Additionally, there was a widening of the gaps among longitudinal muscles, likely attributed to the cut's impact, possibly leading to necrosis. After more than 17 days, group II showed normal architecture and intact nature of circular and longitudinal muscles. Interestingly, group III showed significant wound healing features after three days while group IV took five days and showed less improvement than group III. Semithin section of a group V of *A. caliginosa* revealed the loss of architecture and showed a tendency to develop an excess of glandular epithelium with the disintegration of the cuticular membrane, ectodermal layer, and expansion of spaces between the longitudinal muscles. The neighboring cells of circular and longitudinal muscles showed proliferation of glandular cells erosion in the ectodermal layer of the body wall and discontinuation of cells (separated by narrow to large gap junctions). It may be due to necrosis depending upon the effect of the cut (Fig. 8).

**Fig. 9:**
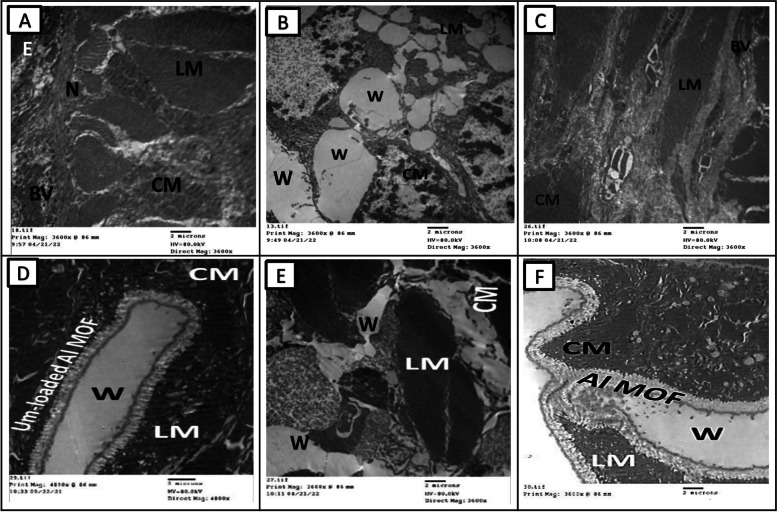
Transmission electron microscopy micrographs of the different groups of earthworms (*Allolobophora caliginosa*) after induction of surgical wounds and examination of wound healing; (**A**) Group I: normal earthworm, (**B**) worm on the first day of injury, (**C**) Group II; worms received Vaseline; (**D**) Group III; worms received Um-Al MOF, (**E**) Group IV; worms received Um, and (**F**) Group V worms received Al-MOF. ((CM); circular muscle layer, (E); epidermis,, (LM); longitudinal muscle layer,(W);Wound,(BV); Blood vessel, (N); Nucleus)

The TEM micrographs capturing the skin of *A. caliginosa* in the control Group I and Group II after 17 days reveal striking details. Surprisingly, the skin of *A. caliginosa* earthworms treated with Um-Al MOF nanoparticles displayed no significant difference compared to the control group and appeared to Um-Al MOF like tape repair the tissue. The development of capillaries, fibroblasts, and collagen in response to a wound, constituting granulation tissue, was evident. However, clear damage was observed in earthworms from Groups IV and V, with the cuticle initiating degradation, the epidermis showing severe necrosis, and circular muscles undergoing total damage. The intercellular matrix exhibited edema and looseness, facilitating minute vessel extension and the creation of new capillaries. Additionally, the proliferation of fibroblasts was observed in these groups (Fig. 9).

## Discussion

Um-Al MOF nanoparticles were successfully prepared by a hydrothermal method. Due to the electrostatic interaction between the Al-MOF nanoparticles and the Um drug, high LC % and maximum EE % were accomplished. Several factors could influence the drug EE and LC percentages of Um in Al-MOF nanoparticles. These factors include the ratio of Um to Al-MOF, sonication time and intensity, and drying methods. Systematically optimization could identify optimal conditions that maximize both EE and LC. The presented results of XRD and FT-IR of the chosen complex affirmed the drug entrapment in the matrix of Al-MOF nanoparticles. Furthermore, TEM displayed its nanosized shape. The thermal decomposition of the nanoparticles was characterized via TGA. The release behavior of Um from Al-MOF nanoparticles showed controllable drug release continued for more than 7 days; this provides a sustainable release of Um over a long period. The free radical scavenging activity test (Scavenging of DPPH radical) showed enhancement of the antioxidant activity of Um-Al MOF as compared to free Um. Besides, the anti-inflammatory activity of Um-Al MOF showed remarkable improvement by measuring the expression levels of COX2, IL6, NF-kB p65, and TNFα. Previously, Um was shown to inhibit NF-kB experimentally and molecular docking was used to decipher the molecular interaction of Um and the active site of NF-kB. Um was shown to form a hydrogen bond with Arginine 365 in the active site of NF-kB [57]. The results indicate that the loading of Um in Al-MOF nanoparticles improves the bioavailability and bioactivity of Um as an antioxidant and anti-inflammatory agent. Similar observations have been reported previously of the enhancing effect of loading Um on different nanocarriers [58–60]. Also, in the present study, observation by the earthworm skin consisting of; epidermis, circular, and longitudinal muscle layers. Group I (the control) body wall structure is the typical organization. Transverse sections were used to examine vacuolization, epithelial cell hypertrophy, and the degeneration of circular and longitudinal muscle layers for group II which received Vaseline only and group V, which had received Al-MOF nanoparticles there hasn't been a reviled or improved or the epidermal layer during the healing process. The results are consistent with previous studies which showed that the thickness of the circular and longitudinal muscle layers decreased [43, 61]. Previous study showed that the circular and longitudinal muscles of the earthworm contract reciprocally to carry out peristalsis under normal circumstances [62]. Crawling activity is hampered if muscle contractions are not coordinated, and the malicious program displays "fictive locomotion." Under normal conditions, the earthworm's feature of regeneration restores its lost parts, particularly the sections that contain the clitellum. This finding agrees with the previously published studies where they demonstrated that regeneration is a complicated mechanism to repair misplaced or broken frame parts [63–66]. The transverse sections of group III that received Um-Al MOF revealed that the inflammatory cells almost vanished and that the body wall had epidermal, circular, and longitudinal muscle structure, these findings agree with previously published data [43]. In the present investigation, the photomicrograph of the semithin section and ultrastructure of the skin of *A. caliginosa* earthworms in institution III that were acquired did not show any extensive variations from group I. Wound recovery entails a boom of capillaries, fibroblasts, and collagen, forming granulation tissue, this result agrees with previously published data [43, 67, 68]. Typically, this histological study confirms that Um promotes the healing of wounds in the *A. caliginosa* earthworm skin version.

## Conclusion

In summary, this investigation has successfully synthesized Um-Al MOF nanoparticles and demonstrated notable drug encapsulation efficiency and loading capacity. Utilizing various characterization techniques, the integration of Um within the Al-MOF framework was confirmed, emphasizing its nanoscale morphology. The developed nanoparticles exhibited sustained drug release kinetics, potent antioxidant properties, and enhanced anti-inflammatory effects compared to free Um. In vivo studies using earthworm models revealed accelerated wound healing facilitated by Um-Al MOF nanoparticles, as evidenced by histological and ultrastructural analyses. These unique properties of controlled drug release, antioxidant, anti-inflammatory, and regenerative attributes, underscore the versatile and promising nature of Um-Al MOF nanoparticles as a potential phytopharmaceutical nanoparticulate system. Although there are structural similarities in the skin structure between earthworms and humans, there are limitations in using earthworms as a model for wound healing. Earthworms possess a basic innate immune system, whereas humans have a more complex immune response that includes both innate and adaptive immunity. Therefore, further investigation and clinical validation in mammalian models, which more closely mimic human skin structure and healing processes is imperative to unlock their broader applications in pharmaceutical and therapeutic domains.

## Data Availability

No datasets were generated or analyzed during the current study.

## Abbreviations

Al-MOF: Aluminum metal–organic framework
Um: Umbelliferon
Um-Al MOF: Umbelliferon-loaded Al-MOF nanoparticles
FT-IR: Fourier-transform infrared spectroscopy
XRD: X-ray diffractometry
UV–Vis: Ultraviolet–visible spectroscopy
TGA: Thermal gravimetric analysis
TEM: Transmission Electron Microscopy
DPPH: 2,2-Diphenyl-1-picrylhydrazyl
COX2: Cyclooxygenase 2
IL6: Interleukin 6
NF-kB p65: Nuclear factor kappa-light-chain-enhancer of activated B cells
TNFα: Tumor Necrosis Factor alpha
